# Unravelling the paradox of vitamin C research in sepsis

**DOI:** 10.1016/j.ccrj.2025.100134

**Published:** 2025-10-14

**Authors:** Tomoko Fujii, Clive N. May, Yugeesh R. Lankadeva

**Affiliations:** a Department of Intensive Care, Jikei University Hospital, Tokyo, Japan; b Australian and New Zealand Intensive Care Research Centre, Monash University School of Public Health and Preventive Medicine, VIC, Australia; c Florey Institute of Neuroscience and Mental Health, Melbourne, VIC, Australia; d Department of Critical Care, Melbourne Medical School, University of Melbourne, Melbourne, VIC, Australia; e Department of Anaesthesia, Austin Hospital, Heidelberg, VIC, Australia

**Keywords:** Vitamin C, Sepsis, Sodium ascorbate, Research program, Preclinical research, Clinical research

## Abstract

Professor Rinaldo Bellomo’s lasting impact on critical care research stems from his commitment to structured, biologically grounded research programs over isolated studies. His work on vitamin C in sepsis exemplifies this approach. While early enthusiasm grew around combination therapies involving vitamin C, Rinaldo championed a cautious, rigorous, and methodical investigation. He worked closely with collaborators to address key methodological issues, including dosing, stability, and the design of appropriate control groups, which ultimately led to the international VITAMINS trial. This landmark study compared vitamin C, hydrocortisone, and thiamine to hydrocortisone alone in septic shock and found no clinical benefit. Rinaldo embedded a pharmacokinetic substudy to confirm supraphysiological serum vitamin C levels, ensuring biological plausibility of the trial design. Beyond clinical research, he fostered translational research with the Florey Institute using a preclinical sheep model of sepsis. This collaboration uncovered critical mechanisms of septic acute kidney injury and led to the development of mega-dose sodium ascorbate therapy. The program progressed from proof-of-concept to a double-blind pilot randomised trial in septic shock and now underpins a national multicentre phase Ib and II clinical trials. Rinaldo’s legacy is defined by scientific rigour, mentorship, and humility. His visionary, disciplined approach remains a model for impactful research and continues to guide ongoing efforts to advance care for critically ill patients.

## 1 Introduction: building credible research programs in critical care

Professor Rinaldo Bellomo (we will refer to simply as Rinaldo, as we always did) believed that clinical research that gives people any answers properly does not come from isolated experiments or opportunistic studies but from organised research programs built on biological plausibility and clinical validity. His collaboration with a group of people who worked on vitamins for sepsis set an example of this philosophy.

Rather than racing towards a single definitive trial, he insisted we lay the groundwork to clarify what was known, question what was assumed, and systematically design each step to inform the next. For the vitamin C program, he had a conceptual framework of the research cycle and moved through mechanistic and pharmacokinetic clinical research and clinical trials. The works continued informing further research pursuing the answers to improve our patient care.

## 2 Vitamin C for sepsis

Strong interest in vitamin C for sepsis took off in 2017 when a dramatic mortality decrease and rapid resolution of shock were reported when 6 g/day of vitamin C was used in a combination with hydrocortisone and thiamine in patients with severe sepsis or septic shock, leading to many patients with septic shock receiving the combination therapy particularly in the US.^1^

The widespread clinical enthusiasm on the combination therapy had outpaced evidence particularly through social media. In the *Critical Care and Resuscitation* in 2018, Rinaldo demonstrated how to address practical concerns such as vitamin C stability and optimal dosing, collaborating with Carr et al. and McNamara et al.^2^^,^^3^ The journal’s editorial outlined the emerging excitement and the risks of premature adoption to clinical practice, surrounding the vitamin C “cocktail” for sepsis and urged caution, advocating for structured evaluation before pursuing large-scale effectiveness trials.^4^

These works were followed by a 2019 point-of-view article written by both vitamin C and corticosteroid investigators, a collaboration that Rinaldo fostered to strengthen the scientific foundation of the vitamin C research program.^5^ The article posed critical questions about comparisons in the design of ongoing clinical trials at that time particularly whether the control groups in the trials should receive protocolised hydrocortisone to ensure a meaningful comparison.

## 3 Designing a clinical trial on vitamin C for sepsis

Hydrocortisone for septic shock was another work Rinaldo contributed, in which the ADRENAL trial team demonstrated that hydrocortisone shortens the duration of vasopressor dependency in septic shock.^6^ The trial results were published in 2018 and were also accompanied by the updated meta-analysis. Given the best available clinical evidence at that time, the optimal trial design for the vitamin C combination therapy that contains hydrocortisone was to be compared with hydrocortisone, which eventually formed the VITAMINS trial.^7^

The VITAMINS trial was an international, open-label randomised clinical trial conducted in Australia, New Zealand, and Brazil, aiming to determine whether the combination of vitamin C (6 g/day), hydrocortisone, and thiamine improves the time to shock resolution when compared with hydrocortisone alone.^8^ Given that the limited data were available for the effect of the intervention, VITAMINS trial employed vasopressor-free hours up to day 7 as the primary outcome and adapted the sample size twice during the trial using the pooled trial data without opening allocation.^7^ Finally, 216 patients were enrolled into the trial and there was no statistically significant difference between the two groups. The findings in the VITAMINS trial were followed by the ATESS trial^9^ and the VICTAS trial.^10^ These randomised clinical trials did not find clinical benefits from using vitamin C and thiamine for sepsis or septic shock.

As the VITAMINS trial was not designed as a definitive confirmative trial, the physiological effect was adopted as the primary outcome measure. To further explore the validity of the dose of vitamin C, Rinaldo embedded a pharmacokinetic substudy in the VITAMINS trial,^11^ which confirmed that the dose (6 g/day, i.e., 1.5 g every 6 h) of vitamin C was sufficient to achieve a supraphysiological level of serum vitamin C levels. The trial was designed not in isolation but with a view from the existing available evidence and towards the next step.

## 4 Was all vitamin C created equal for sepsis?

This seemingly simple question captured the clinical curiosity and scientific rigour of Rinaldo and a team at the Florey Institute of Neuroscience and Mental Health (The Florey).^12^ At a time when enthusiasm for high-dose vitamin C in critical illness outpaced the evidence, the Florey’s team work with Rinaldo strived to bring both clarity and credibility to a controversial field.

The landmark LOVIT trial—the largest to date investigating intravenous ascorbic acid in sepsis—used 200 mg/kg/day of ascorbic acid over 4 days in 863 critically ill patients. The trial reported an unadjusted increase in the composite outcome of mortality or persistent organ dysfunction at day 28 (risk ratio: 1.21; 95% confidence interval: 1.04–1.40) in the vitamin C group compared with the placebo group.^13^ However, this difference was not statistically significant after adjusting for key covariates (RR: 1.15; 95% confidence interval: 0.90–1.47).^13^ Despite the neutral adjusted findings, the trial raised important questions about safety, formulation, and dosing in this patient population.[14], [15], [16]

Rinaldo’s response was characteristically balanced: concern for patient safety, commitment to scientific rigour, and curiosity about the underlying mechanisms. Prior to the published LOVIT trial, Rinaldo has encouraged a systematic scoping review, led by Yanase et al., to evaluate the safety profile of high-dose vitamin C in critically ill patients.^17^ The review identified anecdotal administration of intravenous vitamin C at doses as high as 150 g in oncology and burns without major reported adverse events.^17^ Yet, the Florey team recognised that the acidic formulation of ascorbic acid—administered in large volumes to patients with metabolic acidosis—may present unique risks in the context of critically ill patients with sepsis.^14^ This led to the development of a pH-neutral formulation of vitamin C: sodium ascorbate.

## 5 A new era of vitamin C research: from bench to bedside

From 2003, Rinaldo collaborated closely with the Florey team to codesign a translational large-animal model of sepsis in sheep, capable of continuous monitoring of cardiovascular and renal physiology. Using this model, they overturned the prevailing dogma that renal hypoperfusion was a prerequisite for septic acute kidney injury (AKI), instead demonstrating that AKI could occur despite preserved or even increased renal blood flow.^18^ In 2009, they identified angiotensin II as a novel therapeutic agent for vasoplegia and improved renal function in septic AKI^19^—work that directly contributed to the development of Giapreza.

Building on this foundation, the Florey team and Rinaldo evaluated the effects of mega-dose sodium ascorbate (150 g) in sheep with established septic AKI. Remarkably, sodium ascorbate restored renal medullary perfusion, reversed AKI, improved urine output and creatinine clearance, and reduced vasopressor requirements.^20^ We extended this work with Rinaldo to the brain, showing that sodium ascorbate rapidly corrected frontal cortical tissue ischaemia, hypoxia, and hyperthermia—improvements that were mirrored by striking behavioural recovery in septic animals.^21^ Upon witnessing the behavioural recovery of a sheep from severe Gram-negative sepsis, Rinaldo eloquently remarked, “This is not resuscitation—it is resurrection”.

True to his belief in translating bench research into clinical care, Rinaldo led a double-blind, placebo-controlled trial evaluating 60 g of intravenous sodium ascorbate in 30 patients with septic shock.^22^ The trial demonstrated safety and feasibility, with early signals of physiological benefit—including improved urine output, reduced noradrenaline requirements, and more rapid reductions in Sequential Organ Failure Assessment scores.^22^ This series of preclinical and clinical studies using mega-dose sodium ascorbate to reverse sepsis-induced multiple organ dysfunction laid the foundation for a national program of phase Ib and II clinical trials, supported by $4.9M in Australian federal government funding, to be conducted across every mainland state and territory (MEGASCORES) over the next 4–5 years.

## 6 Summary

Rinaldo’s impact on vitamin C research for sepsis extended far beyond protocol design or trial conduct. He was a steadying voice in a field vulnerable to hype and stigma, reminding us that mechanistic plausibility, rigorous experimentation, and clinical equipoise must guide our investigations. He challenged us to pursue blue-sky ideas that, though initially heretical, might one day become dogma. As he often said: *“Every dogma began as a heresy”*. Beyond his scientific brilliance, Rinaldo will be remembered for his humility, mentorship, and relentless generosity of spirit. Despite a prodigious publication record and an international standing in critical care medicine,^23^ he remained accessible—to students, junior investigators, and colleagues across disciplines. His contributions to sepsis research, and to medicine more broadly, were grounded not only in intellectual excellence but also in an unwavering commitment to improving patient outcomes. We will miss his friendship, his curiosity, and the ever-stimulating conversations—often peppered with literary references, classical analogies, and the occasional playful aside, sometimes featuring his trademark references to Machiavelli and Dante, whom he, as a proud Italian, quoted with characteristic wit and irony. His legacy lives on in the research programs he inspired, the researchers he mentored, and the questions he compelled us to keep asking. Thank you, and farewell, Rinaldo. Your light continues to burn in all of us.

## Funding

This research did not receive any specific grant from funding agencies in the public, commercial, or not-for-profit sectors.

## CRediT authorship contribution statement

**Tomoko Fujii:** Conceptualization, Writing - Original draft preparation. **Clive N May**: Writing - Reviewing and Editing. **Yugeesh R. Lankadeva:** Conceptualisation, Writing - Original draft preparation.

## Conflict of interest

The authors declare the following financial interests/personal relationships which may be considered as potential competing interests: The VITAMINS trial was supported by an Alfred Research Trusts Small Project Grant, the Austin Intensive Care Trust Fund, and a grant from the Intensive Care Foundation and the Institutional Development Support Program of Unified Health System. Tomoko Fujii had been supported by the Japan Society for the Promotion of Science for the VITAMINS trial and has been supported by Japan Society for the Promotion of Science and Terumo Life Science Foundation for other clinical research programs. Yugeesh R Lankadeva reports financial support and administrative support were provided by The Florey Institute of Neuroscience and Mental Health. Yugeesh R Lankadeva reports a relationship with The Florey Institute of Neuroscience and Mental Health that includes: funding grants. Yugeesh R Lankadeva has patent #PCT/AU2021/050321 pending to The Florey Institute of Neuroscience and Mental Health.
